# Frictional resistance in monocrystalline ceramic brackets with conventional and nonconventional elastomeric ligatures

**DOI:** 10.1186/2196-1042-14-9

**Published:** 2013-05-23

**Authors:** Mariana Bulhoes Galvão, Matteo Camporesi, André Tortamano, Gladys Cristina Dominguez, Efisio Defraia

**Affiliations:** Departamento de Ortodontia, Faculdade de Odontologia da Universidade de São Paulo, Av. Prof. Lineu Prestes, 2227, São Paulo, CEP: 05508-000 Brazil; Department of Orthodontic, University of Florence, Via Ponte di Mezzo 46/48, Florence, 50100 Italy

**Keywords:** Frictional resistance, Esthetic fixed appliances, Orthodontic ligatures

## Abstract

**Background:**

The objective of this study was to compare the frictional forces generated by three types of monocrystalline ceramic brackets coupled with conventional elastomeric ligatures (CEL) and nonconventional elastomeric ligatures (NCEL) during the alignment of apically displaced teeth at the maxillary arch.

**Methods:**

All tests (a total of 480 tests) were carried out in a dry state on a universal testing machine with a testing model consisting of three 0.022-in. monocrystalline ceramic preadjusted brackets (from the maxillary right second premolar through the right central incisor). The canine bracket was bonded to a sliding bar that allowed for different vertical positions. The frictional forces generated by a 0.012- and 0.014-in. superelastic nickel titanium wire (SENT) with conventional and nonconventional ligatures at various amounts of canine misalignment (1.5, 3.0, 4.5, and 6.0 mm) were recorded. Comparisons between the different types of bracket-wire-ligature systems were carried out by means of analysis of variance on ranks with Tukey's *post hoc* test (*P* < 0.05).

**Results:**

No significant differences were assessed among the three types of monocrystalline brackets with NCEL when coupled with 0.012-in. SENT. Radiance brackets with NCEL coupled with 0.014-in. SENT showed significantly greater frictional force than Inspire Ice brackets and Pure brackets with NCEL. A significantly greater amount of frictional force was generated with CEL when compared with NCEL for all the tested variables, with the exception of the Pure brackets with 0.012-in. SENT at 1.5 and 3.0 mm of canine misalignment where similar frictional forces were found.

**Conclusions:**

Nonconventional elastomeric ligatures are able to reduce friction in monocrystalline ceramic brackets.

## Background

In modern society, the esthetic aspect of orthodontic therapy is becoming increasingly important because of the growing number of adult patients. Ceramic brackets have been developed to improve esthetics during orthodontic treatment [1, 2]. However, it has been observed both clinically and with experimental *in vitro* studies that the efficiency of tooth movement during sliding mechanics with ceramic brackets is significantly lower than that shown by metal brackets [3, 4].

All currently available ceramic brackets are composed of aluminum oxide. Because of their distinct differences during fabrication, two types of ceramic brackets are available, namely the polycrystalline alumina and the single crystal alumina or monocrystalline alumina [5]. The most apparent difference between polycrystalline and single crystal brackets is in their optical clarity. Single crystal brackets are noticeably clearer than polycrystalline brackets, which tend to be translucent and more esthetic [6]. It has been reported that under all conditions tested, ceramic brackets generate higher frictional forces than stainless steel brackets. Despite their superior esthetics, monocrystalline ceramic brackets produce higher frictional forces than polycrystalline ceramic brackets [7, 8]. Nonconventional elastomeric ligatures (NCEL) have been proposed to reduce frictional resistance in treatment mechanics with preadjusted fixed appliances. These ligatures can be used on any type of conventional brackets, esthetic brackets included [9–12]. The aim of this *in vitro* study was to analyze the frictional forces released during the leveling and aligning phase of fixed appliance therapy by three types of monocrystalline ceramic brackets with conventional elastomeric ligatures (CEL) compared with nonconventional elastomeric ligatures (NCEL) at different amounts of vertical canine misalignment.

## Methods

**Figure 1:**
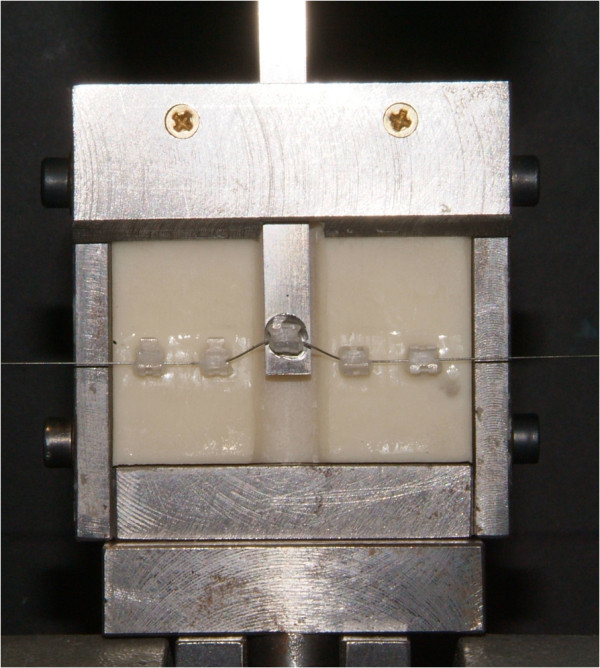
**Experimental** ***in vitro*** **model with misaligned canine bracket.**

An experimental model [11] (Figure 1) was used to assess the frictional forces produced by three types of 0.022-in. slot monocrystalline ceramic preadjusted brackets with Roth prescription combined with different types of elastomeric ligatures:

- Monocrystalline ceramic brackets (Inspire Ice, Ormco, Orange, CA, USA) with CEL (Unisticks Clear, American Orthodontics, Sheboygan, WI, USA)
- Monocrystalline ceramic brackets (Inspire Ice, Ormco) with esthetic NCEL (Slide AQUA, Leone Orthodontic Products, Sesto Fiorentino, Firenze, Italy)
- Monocrystalline ceramic brackets (Radiance, American Orthodontics) with esthetic CEL (Unisticks Clear, American Orthodontics)
- Monocrystalline ceramic brackets (Radiance, American Orthodontics) with esthetic NCEL (Slide AQUA, Leone Orthodontic Products)
- Monocrystalline ceramic brackets (Pure, Ortho Technology, Tampa, FL, USA) with esthetic CEL (Unisticks Clear, American Orthodontics)
- Monocrystalline ceramic brackets (Pure, Ortho Technology) with esthetic NCEL (Slide AQUA, Leone Orthodontic Products)

The buccal segment model consisted of five (from the second premolar to the central incisor) monocrystalline ceramic brackets [11]. The interbracket distance was set at 8.5 mm. The canine bracket was welded to a sliding bar that allowed different vertical positions, while the other brackets were mounted on a vice-like device. A section of a 0.0215 × 0.028-in. stainless steel wire was used to align all the brackets.

Two sizes of round superelastic nickel titanium wires (SENT) (Memoria wire, Leone Orthodontic Products), 0.012 and 0.014 in., were tested with the different combinations of esthetic brackets and elastomeric ligatures at different amounts of apical canine misalignment (1.5, 3.0, 4.5, and 6.0 mm). Both NCEL and CEL were placed immediately before each test run to prevent ligature force decay.

**Figure 2:**
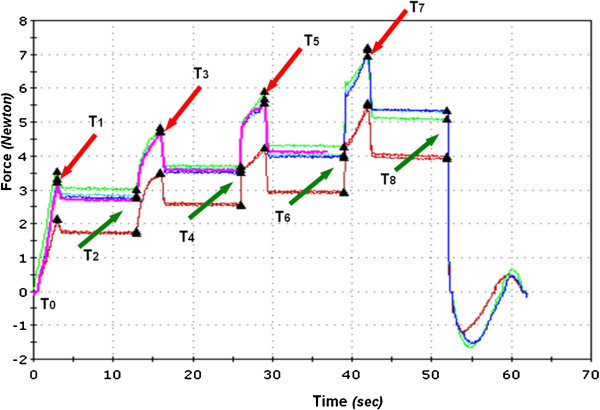
**Graphical representation of the forces analyzed.** The different colored lines in the graph represent the four tests.

**Figure 3:**
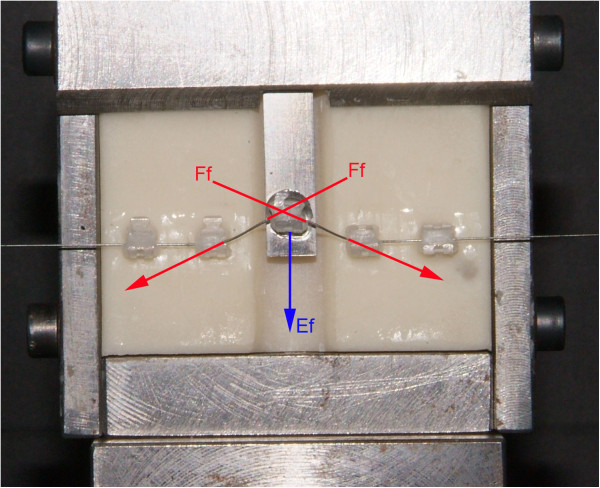
**In the upward movement, the Instron machine analyzes the frictional forces (** ***F***_**f**_ **) and elastic forces (** ***E***_**f**_ **).**

**Figure 4:**
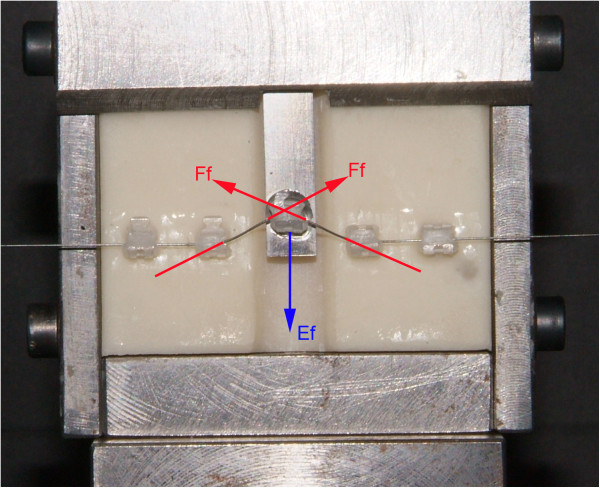
**When movement is stopped, the Instron machine analyzes the same forces, but** ***F*** **f shows opposite direction.**

The forces developed by the testing unit consisting of wire, brackets, and elastomeric ligatures were measured under dry conditions and at room temperature (20°C ± 2°C) by means of an Instron 3365 testing machine (Instron Corp., Canton, MA, USA) with a load cell of 10 N. The upper end of the sliding bar bearing the canine bracket was connected to the Instron crosshead. The frictional forces were calculated as reported in the ‘Appendix’ (Figures 2, 3, 4). The frictional forces generated by each bracket-wire-ligature combination at the different amounts of vertical canine misalignment were tested ten times with new wires and new ligatures for each test. A total of 480 tests (240 tests with NCEL and 240 tests with CEL) were carried out.

### Statistical analysis

Descriptive statistics were calculated for the frictional forces produced by the different bracket-wire-ligature combinations. Because normal distribution of the data was not found (Shapiro-Wilk test), the comparisons were carried out using a nonparametric test. Statistical between-group comparisons were performed by means of analysis of variance (ANOVA) on ranks (Kruskal-Wallis tests) with Tukey's *post hoc* tests. The level of significance for all tests was set at *P* < 0.05. All statistical computations were performed using a statistical software (SAS 8.0, SAS Institute Inc., Cary, NC, USA).

## Results

The descriptive statistics and statistical comparisons of the frictional forces generated by the monocrystalline ceramic brackets are shown in Tables 1 and 2. All brackets with NCEL produced significantly lower frictional forces than the CEL at all amounts of canine misalignment with both 0.012- and 0.014-in. SENT wires. The only exceptions were at 1.5 and 3.0 mm of canine displacement with the 0.012-in. SENT where the NCEL on Pure brackets was not significantly different from the CEL.

**Table 1:** **Descriptive statistics for the frictional forces (Newton)**

SENT (in.)	CM (mm)	Inspire Ice-CEL		Inspire Ice-NCEL		Radiance-CEL		Radiance-NCEL		Pure CEL		Pure NCEL	
		Mean	SD	Mean	SD	Mean	SD	Mean	SD	Mean	SD	Mean	SD
0.12	1.5	0.177	0.028	0.045	0.005	0.147	0.021	0.029	0.013	0.100	0.025	0.059	0.011
	3.0	0.279	0.016	0.178	0.021	0.361	0.026	0.187	0.026	0.248	0.038	0.184	0.014
	4.5	0.362	0.033	0.212	0.025	0.467	0.041	0.279	0.054	0.327	0.031	0.236	0.026
	6.0	0.474	0.108	0.256	0.025	0.510	0.026	0.290	0.042	0.415	0.049	0.301	0.028
0.14	1.5	0.175	0.018	0.088	0.006	0.206	0.020	0.095	0.016	0.145	0.019	0.062	0.015
	3.0	0.335	0.013	0.226	0.009	0.471	0.020	0.318	0.014	0.331	0.010	0.214	0.005
	4.5	0.429	0.018	0.275	0.017	0.647	0.063	0.441	0.041	0.444	0.011	0.323	0.047
	6.0	0.496	0.024	0.354	0.027	0.773	0.113	0.481	0.106	0.422	0.056	0.348	0.028

*CM* canine misalignment, *SENT* superelastic nickel titanium wire, *CEL* conventional elastomeric ligatures, *NCEL* nonconventional elastomeric ligatures.

**Table 2:** **Statistical between-group comparisons (ANOVA on ranks with Tukey's** ***post hoc*** **tests)**

SENT (in.)	CM (mm)	***P*** value								
		Inspire Ice-CEL vs Inspire Ice-NCEL	Radiance-CEL vs Radiance-NCEL	Pure CEL vs Pure NCEL	Inspire Ice-CEL vs Pure CEL	Inspire Ice-CEL vs Radiance-CEL	Radiance-CEL vs Pure CEL	Inspire Ice-NCEL vs Pure NCEL	Inspire Ice-NCEL vs Radiance-NCEL	Radiance-NCEL vs Pure NCEL
0.12	1.5	<0.001	<0.001	0.955	0.004	>0.999	0.781	>0.999	>0.999	>0.999
	3.0	<0.001	<0.001	0.093	>0.999	0.001	<0.001	>0.999	>0.999	>0.999
	4.5	<0.001	<0.001	<0.001	0.999	<0.001	<0.001	>0.999	0.046	0.915
	6.0	<0.001	<0.001	<0.001	0.225	0.995	<0.001	0.884	0.999	>0.999
0.14	1.5	<0.001	<0.001	0.001	>0.999	>0.999	0.138	>0.999	>0.999	0.999
	3.0	<0.001	<0.001	<0.001	>0.999	<0.001	<0.001	>0.999	<0.001	<0.001
	4.5	<0.001	<0.001	<0.001	>0.999	<0.001	<0.001	0.745	<0.001	<0.001
	6.0	<0.001	<0.001	0.008	0.009	<0.001	<0.001	>0.999	<0.001	<0.001

*CM* canine misalignment, *SENT* superelastic nickel titanium wire, *CEL* conventional elastomeric ligatures, *NCEL* nonconventional elastomeric ligatures.

When coupled with 0.012-in. SENT, the Inspire Ice brackets with CEL showed a significantly greater frictional force when compared with the Pure brackets with CEL at 1.5 mm. No differences were found between the Inspire Ice brackets with CEL and Radiance brackets with CEL and between the Radiance brackets with CEL and Pure brackets with CEL at 1.5 mm. With the same wire, the Radiance brackets with CEL showed a significantly greater frictional force when compared to all other brackets with CEL at 3.0 and 4.5 mm. At 6.0 mm of canine misalignment, the Radiance brackets with CEL generated a frictional force similar to that of the Inspire Ice brackets with CEL but significantly greater than that produced by the Pure brackets with CEL. When coupled with a 0.014-in. SENT, the Radiance brackets with CEL showed significantly greater frictional forces at all amounts of canine misalignment when compared with the Inspire Ice brackets and Pure brackets with CEL, with the exception of the 1.5 mm canine displacement where no differences were observed.

No significant differences were assessed among the three types of monocrystalline ceramic brackets with NCEL using a 0.012-in. SENT. With a 0.014-in. SENT, the Radiance brackets with NCEL showed a significantly greater frictional resistance than the Inspire Ice brackets and Pure brackets with NCEL at all amounts of canine displacements, with the exception of the 1.5 mm canine misalignment where no significant differences were found.

## Discussion

Friction has been studied in a number of ways. In some studies, the wires were pulled through at least one bracket; in other instances [4], a bracket was slid on a wire. Laboratory tests are usually simplified and designed to look at only one or two variables related to the wires tested, and that makes generalization difficult. The bracket-wire interface varies significantly according to the ligation mechanism used [8, 11, 13–18].

Clinicians are not generally interested in knowing the coefficient of friction for a specific type of wire when used with a specific bracket or how much of the resistance to sliding results from friction versus binding. Clinicians need to know the forces applied on the dentition when specific combinations of bracket, wire, and ligation method are used in a malocclusion [14].

In this study, the frictional forces generated by the bracket-wire-ligature system with three types of monocrystalline ceramic brackets with CEL and NCEL during the leveling phase of fixed appliance therapy were analyzed. A testing device similar to the one proposed by Franchi and Baccetti [11] was conceived to re-create clinical conditions for the leveling and aligning phase of the straight-wire technique, i.e., to study the frictional forces generated during the leveling of a displaced tooth by allowing different amounts of vertical misalignment of one bracket (canine bracket) with respect to the four remaining aligned brackets.

Franchi and Baccetti in 2006 [11] observed that when a slight amount of tooth alignment is needed (1.5 mm), differences in the performance of conventional and low-friction ligatures were minimal, but they became significant for correction of a misalignment greater than 3.0 mm. In 2007, Camporesi et al. [12] evaluated the forces available for tooth alignment in the presence of preadjusted 0.022-in. polycrystalline ceramic brackets coupled with low-friction esthetic ligatures and confirmed what had been found with metal brackets in 2006 [11].

In the current study, all monocrystalline ceramic brackets with NCEL produced significantly lower frictional forces than CEL at all amounts of canine misalignment. The results of the present study agree also with those reported by Franchi and Baccetti [11] and Camporesi et al. [12]. It should be noted, however, that the present study measured the frictional forces developed by the bracket-wire-ligature system rather than the forces available for tooth movement (see ‘Appendix’) [11, 12]. The results reported here are also consistent with those of Tecco et al. [13] who found that NCEL showed lower friction when compared with conventional ligatures when coupled with round archwires.

In the current study, no significant differences were assessed among the three types of monocrystalline ceramic brackets when used in combination with NCEL and 0.012-in. SENT wire. In the presence of CEL and 0.012-in. SENT wire, the Inspire Ice brackets showed a significantly greater frictional force when compared with the Pure brackets at 1.5 mm, while the Radiance brackets developed significantly greater frictional forces than all other esthetic brackets at 3.0 and at 4.5 mm. At 6.0 mm of canine displacement, the Radiance brackets with CEL and 0.012-in. SENT wire showed frictional forces similar to those of the Inspire Ice brackets but greater than those of the Pure brackets.

The present study also demonstrated that in the presence of either type of elastomeric ligature coupled with 0.014-in. SENT wire, the Radiance brackets showed significantly greater frictional forces when compared with the other esthetic brackets at all amounts of canine displacement with the exception of 1.5 mm canine misalignment where no significant differences were found.

Obviously, the advantage of an *in vitro* study such as this is that confounding effects and extrinsic variables are more easily minimized, but there are certain limitations of this study. In particular, the clinical interpretation of these data requires further considerations that modulate these findings. First, the vice-like devices of the testing machine did not allow the brackets contiguous to the misaligned bracket to move. Our results, therefore, relate to a condition that can be defined as absolute anchorage. Second, each test with the machine was performed with new elastomeric ligatures. No attempt was made to evaluate the effects of time and oral environment on the amounts of force released with the different types of elastomeric ligatures [19].

## Conclusions

The present experiment emphasized the efficiency of the nonconventional ligatures in low-friction esthetic systems. Nonconventional ligatures showed lower friction when compared with conventional ligatures when coupled with round nickel titanium superelastic archwires in monocrystalline ceramic brackets.

## Appendix

The forces generated by the system are recorded at the following steps (Figure 2):

T0: Start of the test. The bracket for the canine is correctly aligned.

T1: At 1.5 mm of canine misalignment (after 3 s). Peak force at 1.5 mm

T2: At 1.5 mm of canine misalignment (after 10 s from T1 and 13 s from T0). Plateau force at 1.5 mm

T3: At 3.0 mm of canine misalignment (after 3 s from T2 and 16 s from T0). Peak force at 3.0 mm

T4: At 3.0 mm of canine misalignment (after 10 s from T3 and 26 s from T0). Plateau at 3.0 mm

T5: At 4.5 mm of canine misalignment (after 3 s from T4 and 29 s from T0). Peak force at 4.5 mm

T6: At 4.5 mm of canine misalignment (after 10 s from T5 and 39 s from T0). Plateau at 4.5 mm

T7: At 6.0 mm of canine misalignment (after 3 s from T6 and 42 s from T0). Peak force at 6.0 mm

T8: At 6.0 mm of canine misalignment (after 10 s from T7 and 52 s from T0). Plateau at 6.0 mm

The bracket for the canine concludes the cycle going back to 0 mm (0 to 6.0 mm in four steps: 1.5, 3.0, 4.5, and 6.0 mm and goes back to 0 mm); see Figure 2. Schematic representation of the experimental model used to assess the frictional forces was produced (Figures 3 and 4).

To examine the magnitude of *F*_f_ and *E*_f_ knowing the magnitude of *F*_1_ and *F*_2_, the following equation system was solved:where *F*_f_ are the frictional forces, *E*_f_ are the elastic forces, *F*_1_ are the forces at the peak, and *F*_2_ are forces analyzed at the plateau end.
